# A core microbiome associated with the peritoneal tumors of pseudomyxoma peritonei

**DOI:** 10.1186/1750-1172-8-105

**Published:** 2013-07-12

**Authors:** Jeremy J Gilbreath, Cristina Semino-Mora, Christopher J Friedline, Hui Liu, Kip L Bodi, Thomas J McAvoy, Jennifer Francis, Carol Nieroda, Armando Sardi, Andre Dubois, David W Lazinski, Andrew Camilli, Traci L Testerman, D Scott Merrell

**Affiliations:** 1 Department of Microbiology and Immunology, Uniformed Services University of the Health Sciences, Bethesda, MD 20814, USA; 2 Department of Medicine, Uniformed Services University of the Health Sciences, Bethesda, MD 20814, USA; 3 Center for the Study of Biological Complexity, Virginia Commonwealth University, Richmond, VA 23284, USA; 4 Tufts University Core Facility, Tufts University, Boston, MA 02111, USA; 5 Department of Chemical and Biomolecular Engineering, University of Maryland, College Park, MD 20742, USA; 6 Mercy Health Services, Baltimore, MD 21202, USA; 7 Department of Molecular Biology and Microbiology, Tufts School of Medicine, Boston, MA 02111, USA; 8 Howard Hughes Medical Institute, Boston, MA 02111, USA; 9 Department of Microbiology and Immunology, Louisiana State University Health Sciences Center-Shreveport, Shreveport, LA 71130, USA

**Keywords:** PMP, Pseudomyxoma peritonei, Peritoneal cancer, Microbiome, Microbiota

## Abstract

**Background:**

Pseudomyxoma peritonei (PMP) is a malignancy characterized by dissemination of mucus-secreting cells throughout the peritoneum. This disease is associated with significant morbidity and mortality and despite effective treatment options for early-stage disease, patients with PMP often relapse. Thus, there is a need for additional treatment options to reduce relapse rate and increase long-term survival. A previous study identified the presence of both typed and non-culturable bacteria associated with PMP tissue and determined that increased bacterial density was associated with more severe disease. These findings highlighted the possible role for bacteria in PMP disease.

**Methods:**

To more clearly define the bacterial communities associated with PMP disease, we employed a sequenced-based analysis to profile the bacterial populations found in PMP tumor and mucin tissue in 11 patients. Sequencing data were confirmed by *in situ* hybridization at multiple taxonomic depths and by culturing. A pilot clinical study was initiated to determine whether the addition of antibiotic therapy affected PMP patient outcome.

**Main results:**

We determined that the types of bacteria present are highly conserved in all PMP patients; the dominant phyla are the Proteobacteria, Actinobacteria, Firmicutes and Bacteroidetes. A core set of taxon-specific sequences were found in all 11 patients; many of these sequences were classified into taxonomic groups that also contain known human pathogens. *In situ* hybridization directly confirmed the presence of bacteria in PMP at multiple taxonomic depths and supported our sequence-based analysis. Furthermore, culturing of PMP tissue samples allowed us to isolate 11 different bacterial strains from eight independent patients, and *in vitro* analysis of subset of these isolates suggests that at least some of these strains may interact with the PMP-associated mucin MUC2. Finally, we provide evidence suggesting that targeting these bacteria with antibiotic treatment may increase the survival of PMP patients.

**Conclusions:**

Using 16S amplicon-based sequencing, direct *in situ* hybridization analysis and culturing methods, we have identified numerous bacterial taxa that are consistently present in all PMP patients tested. Combined with data from a pilot clinical study, these data support the hypothesis that adding antimicrobials to the standard PMP treatment could improve PMP patient survival.

## Background

Pseudomyxoma peritonei (PMP) is a clinical syndrome characterized by the dissemination of mucus-secreting tumors throughout the peritoneal cavity [1-3]. This rare but devastating condition usually arises after rupture of a mucin-producing appendiceal neoplasm and is associated with significant morbidity. Mortality usually results from diffuse spread of mucin within the peritoneum and mechanical obstruction of the intestines. PMP is generally classified into two major categories: diffuse peritoneal adenomucinosis (DPAM) and peritoneal mucinous adenocarcinoma (PMCA) [3-5]. DPAM is the lower grade of PMP disease and refers to an indolent tumor form without cellular atypia and with no invasive features. In contrast, PMCA is characterized by severe cellular atypia, poor histologic cellular differentiation and tissue invasion. When proper treatment is given in a timely manner, the prognosis for patients with DPAM can be good (five year survival rate of ~75%); however, patients with the more severe PMCA do not fare as well (five year survival rate of ~14%) [6]. Current therapeutic strategies consist of macroscopic tumor and mucin removal by cytoreductive surgery followed by hyperthermic intraperitoneal chemotherapy [3,5,7,8]. However, despite aggressive treatment, the disease frequently relapses and the ten year survival is only 32% [1,9,10]. As such, there is a great need for ways to improve long-term patient outcome.

Within the past several years, it has become increasingly clear that the microbial communities in and on the human body have a far-reaching impact on human physiology and serve to maintain the balance between health and disease states [11-21]. More recently these interactions have been linked to human cancers [12,17] and have even led researchers to identify particular bacterial taxa that are enriched in tumor environments compared to surrounding tissue [12,17]. These insights not only provide novel information about tumor development and subsequent disease progression, but also highlight potential treatment options for these cancers. Using probes designed to recognize both typed and non-culturable bacteria (TNCB), as well as the known bacterial carcinogen *Helicobacter pylori*, a recent study observed that there are numerous types of bacteria, which are associated with the PMP tumors and secreted mucin within the normally sterile peritoneal cavity [10]. In PMP tissue, the density of TNCB and *H*. *pylori* was highest in the more malignant form of PMP; however, the identity of these TNCB was not determined. Taken together, those results suggested that PMP disease progression may be associated with the presence of bacteria within the peritoneum.

We hypothesize that bacteria play a role in the progression or maintenance of PMP disease. In the current study, we utilized high-throughput sequencing of amplicons from the V6 hyper-variable region of the 16S ribosomal RNA (rRNA) gene to characterize the bacterial communities associated with tumors and secreted mucin in 11 PMP patients. We identified a highly conserved core group of bacterial taxa that were found in all patients. The core community membership was consistent across all patients tested, although the relative abundance of these bacteria varied from patient to patient. Specific DNA probes and *in situ* hybridization (ISH) was used to directly detect PMP community members across a range of taxonomic depths. Furthermore, multiple bacterial isolates were obtained by culturing PMP tissue samples under microaerophilic conditions; some of these isolates interact with the PMP-associated mucin MUC2 as well as host cells *in vitro*. Based on these and previous findings [10], we initiated a pilot clinical study to evaluate the possibility of using antibiotic therapy to target these bacterial communities in the treatment of PMP. Consistent with the hypothesis that these bacteria play a role in PMP disease, our preliminary clinical findings suggest that treating PMP patients with antibiotics early in disease progression may increase long-term survival.

## Methods

### PMP patients

This study utilized stored PMP patient specimens that were collected peri-operatively at Mercy Medical Center in Baltimore, MD. Tissue was aseptically collected in a sterile surgical suite and stored at −80°C until the samples were ready to process. Free mucin was collected from the peritoneal cavity, and tumor tissue was biopsied from the peritoneal wall; by following these guidelines, the two sample types are visually distinguishable from one another. All procedures were approved by the IRB at all centers involved in this study. Written informed consent was obtained from all patients prior to enrollment in the study.

The study was in compliance with the Helsinki Declaration and was approved by the Institutional Review Boards of the Mercy Medical Center and the Uniformed Services University of the Health Sciences. Written informed consent was obtained from all patients before study entry.

### DNA extraction, V6 PCR and sequencing

Total genomic DNA was isolated from PMP tissue specimens and stored at -80C. Between 100-200 ng of genomic DNA was used as template for PCR with a combination of V6-specific primers as previously described [22,23]. The products of 2–3 amplifications from each sample were gel purified and pooled. For each set of amplifications, a no template control was included to ensure that amplification was not the result of DNA contamination. Additionally, reagents used for DNA elution were tested for contaminating bacterial DNA by PCR. Pooled amplicons were barcoded, combined into a multiplexed sample and sequenced on an Illumina HiSeq2000 at the Tufts University Core Facility. Raw reads were de-multiplexed, filtered for quality and barcodes/adapters were removed using Galaxy [24].

### V6 sequence processing and analysis

All subsequent sequence analyses were performed using mothur [25]. To simplify the dataset, each sample was randomly sub-sampled to a depth of 100,000 reads per sample. Sequences were aligned to a V6-specific, curated database [22] derived from the full-length SILVA alignment distributed with mothur. The alignment was screened to remove sequences that were shorter than 56 bp or greater than 72 bp in length as well as any sequences that contained an ambiguous base call or homopolymer runs of >4 bp. The resulting alignment was then filtered to remove any columns that contained missing information. Reads were pre-clustered so that any sequences that contained a single base pair difference were considered to be the same. Pre-clustered reads were classified using the mothur implementation of the RDP Bayesian classifier [26] using a cutoff of 60% bootstrap support over 100 iterations. Sequences that were classified as mitochondria, cyanobacteria, chloroplasts and sequences that were classified as “unknown,” or “unclassified” at the phylum level were removed from the dataset. The remaining aligned sequences were used to generate a pairwise distance matrix and clustered into operational taxonomic units (OTUs) using average-linkage clustering at an identity of 97%. OTUs were classified using the mothur implementation of the RDP classifier as described above. Prior to α- and β-diversity calculations the sequences from each sample were normalized based on the lowest number of OTUs found in any of the samples. As indicated by Good’s coverage estimate, this workflow provided ample sample coverage (range of coverage was 0.96 to 0.99, with a mean coverage of 0.976).

### *In situ* hybridization (ISH) studies

16S and 23S rRNA-specific probes used for ISH are listed in Table 1. The Actinobacteria, Bacteroidetes, Betaproteobacteria, Gammaproteobacteria, Firmicutes, Rhizobiales, *Pseudomonas*, *Streptococcus* and Verrucomicrobiales probe sequences were obtained from probeBase (retrieved from: http://www.microbial-ecology.net/probebase). The *Propionibacterium*-specific probe was designed using 47 randomly selected full-length *Propionibacterium* 16S sequences obtained from the RDP database. The specificity of the probe sequence was verified using the ProbeMatch function on the RDP website; the probe sequence used detects ~97% of sequences classified into the *Propionibacterium* genus. Labeling and hybridization procedures were performed as previously described [10]. Briefly, formalin-fixed tissue blocks were cut into 5 micron sections; each unstained section was deparaffinized, pre-hybridized and subsequently treated with denatured probe solution for 18 h at 37°C. The hybridization mixture contained one of the 10 different biotinylated taxa-specific probes. After hybridization, unbound probe was removed by successive washes in decreasing concentrations of SSC (2× SSC for 30 min, 1× SSC for 10 min, 0.5× SSC for 10 min and 0.1× SSC for 15 min at 60°C). Probes were detected using a streptavidin conjugated with fluorescein (FITC). To ensure that the FITC-conjugated secondary did not interact with the tissue non-specifically and to control for background fluorescence, we performed control hybridizations without the addition of the primary probe. Sections were mounted with Vectashield (Vector Labs, Burlingame, CA), and reactions were observed using a Nikon Eclipse 80i microscope with a DS camera, a DS-L2 control unit and an NIS-Elements scope. Control hybridizations for nonspecific binding were performed for all probes used in this study.

**Table 1:** ISH probes used in this study

**Probe (Abbreviation)**	**Taxonomic depth**	**Sequence (5’-3’)**	**Target**
Actinobacteria (ACT)	Phylum	TATAGTTACCACCGCCGT	23S rRNA
Bacteroidetes (BAC)	Phylum	AGCTGCCTTCGCAATCGG	16S rRNA
Firmicutes (FIR)	Phylum	TGGAAGATTCCCTACTGC	16S rRNA
Betaproteobacteria (BET)	Class	CCCATTGTCCAAAATTCCCC	16S rRNA
Gammaproteobacteria (GAM)	Class	GCCTTCCCACATCGTTT	16S rRNA
Rhizobiales (RHI)	Order	TCGCTGCCCACTGTCACC	16S rRNA
Verrucomicrobiales (VER)	Order	GCTGCCACCCGTAGGTGT	16S rRNA
Pseudomonas (PSE)	Genus	TCTGGAAAGTTCTCAGCA	16S rRNA
Propionibacterium (PRO)	Genus	CACCCATCTCTGAGCACCCCG	16S rRNA
Streptococcus (STR)	Genus	CCACTCTCCCCTTCTGCAC	16S rRNA

### Culturing and identification of bacterial isolates from PMP tissue

Aseptically collected surgical specimens were shipped overnight on ice to the LSU Health Sciences Center Shreveport. As *H*. *pylori* was previously identified in PMP tissue [10], initial culture conditions were chosen to be suitable for cultivating this organism. Upon receipt, amounts of material weighing up to 1 gram were placed in microcentrifuge tubes containing 2 mm zirconium oxide beads and homogenized for four minutes using a Bullet Blender (Next Advance, Inc., Averill Park, NY). Twenty-five to two hundred microliters of each homogenate was added to 25 cm^2^ tissue culture flasks containing 5 ml Ham’s F-12 Nutrient Mixture supplemented with 2% fetal bovine serum (fbs), 12.5 μg/ml nicotinamide adenine dinucleotide (NAD), and 3 μg/ml cytochalasin B. Cytochalasin B was used to inhibit phagocytosis of bacteria by macrophages. Flasks were incubated in microaerobic conditions, monitored daily for growth and sub-cultured once growth was observed. Each isolate was serially passaged multiple times until the culture was axenic. Full-length 16S rRNA gene sequences were PCR amplified using FideliTaq high fidelity polymerase (USB) and the 8F and 1492R primers; amplicons were sub-cloned into pGEM-T Easy (Promega). Cloned 16S rRNA genes were sequenced with 1492R and 907R primers. Sequences were classified using the web-based RDP Bayesian classifier (http://rdp.cme.msu.edu) and the RDP training set 9. Adherence assays were performed by co-culturing selected isolates with HT-29 cells, which produce the PMP-associated mucin MUC2. Interaction with MUC2 was detected by immunohistochemistry. Briefly, *in vitro* MUC2 production was detected using a polyclonal anti-MUC2 antibody; the MUC2::MUC2 antibody conjugates were detected using biotinylated anti-rabbit antibody and avidin-peroxidase. To ensure that the signal detected was specific to MUC2, we also performed control hybridizations with non-MUC2 secreting cells, as well as MUC2 secreting cells in the absence of bacteria.

### Clinical trial study design

A pilot clinical study was initiated to evaluate the possible association between bacteria and the development of PMP disease and to determine the benefit of antibiotic treatment. Beginning in September of 2007, a group of 21 patients with PMP was treated with antibiotics in conjunction with the standard cyroreduction surgery and hyperthermic intraperitoneal chemotherapy treatment with mitomycin C. The treatment group was comprised of eight patients with DPAM, 11 with PMCA, one patient with both diagnoses and one patient with no available diagnosis. Patients were treated with Prevpac®, which includes 30 mg lansoprazole, 500 mg amoxicillin, and 500 mg clarithromycin; For each course of treatment, antibiotics were taken twice daily for 10–14 days. Lymph node status was determined by a pathologist at Mercy Medical Center, Baltimore, MD; positive lymph nodes were identified by the presence of cancer cells. Patient survival was compared using a log-rank statistical test.

## Results

### Sequence analysis and identification of a core PMP Microbiome

A previous ISH-based study identified the presence of many typed and non-culturable bacteria in tissue samples taken from PMP tumors and secreted mucin [10]. This study highlighted the possibility that these bacteria play a role in PMP disease progression; thus, these bacteria could be targeted with antibiotics as a novel, ancillary therapeutic option. In order to investigate the presence of these bacteria further, we collected paired tissue samples from 11 PMP patients (11 tumor and 11 mucin samples = 22 total) that had not been given antibiotic treatment, and used a V6-based analysis to profile the bacterial communities associated with both tumor and secreted mucin.

**Figure 1:**
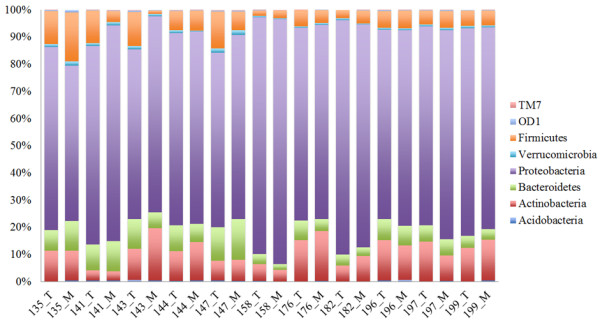
**Distribution of prominent bacterial phyla.** The relative abundance of each phylum is shown as the number of OTUs that were classified in each phylum out of the total number of bacterial OTUs. Numbers on the X-axis represent patient designations. “T” indicates the sample was taken from tumor tissue and “M” indicates the sample was taken from mucin.

After clustering sequences into OTUs at a level of 97%, we first examined the community profiles at the phylum level. As shown in Figure 1, the distribution of bacterial phyla between samples was consistent; the most prominent phylum represented in all samples was the Proteobacteria. The relative abundance of the Proteobacteria across the 22 samples ranged from 56.8% to 90.2%, with a mean of 73.0%. Other prominent phyla include the Actinobacteria (3.4% to 19.3%, mean of 10.7%), Firmicutes (1.3% to 17.8%, mean of 6.9%), Bacteriodetes (2.0% to 14.8%, mean of 7.2%), Verrucomicrobia (0.3% to 1.7%, mean of 0.8%), Acidobacteria (0% to 0.6%, mean of 0.3%), TM7 (0% to 0.3%, mean of 0.1%) and OD1 (0% to 0.9%, mean of 0.2%).

**Figure 2:**
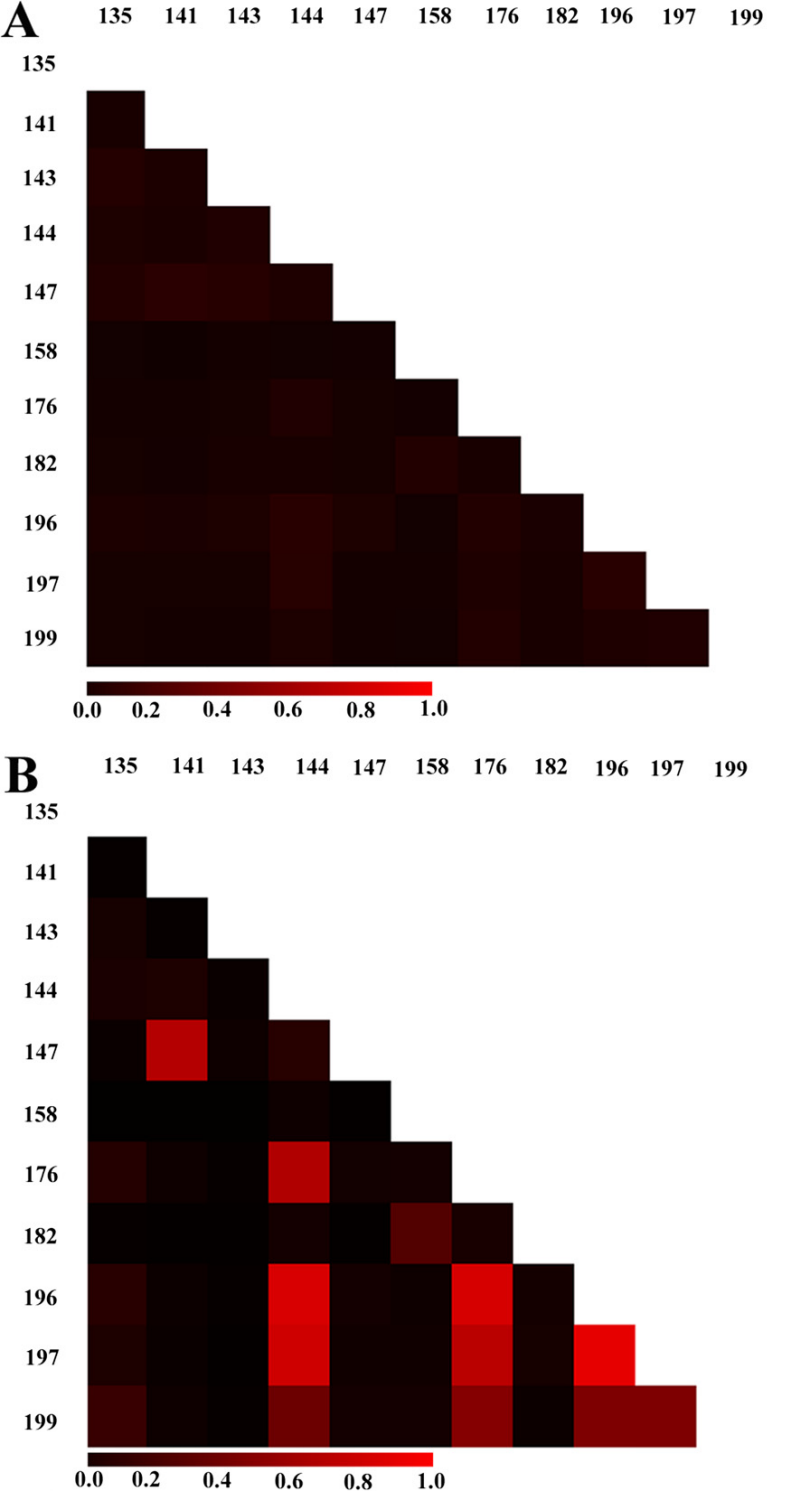
**β-diversity between PMP communities.** Heatmaps compare the classic Jaccard (Jclass) dissimilarity indices **(A)** and thetaYC indices **(B)** for bacterial communities found in PMP patients. Numbering along the left-hand and top sides of each heatmap represent individual patient designations. Each square within the heatmap represents a single pairwise comparison between the corresponding patient communities (labeled at the top and left side of each heatmap). As indicated by the bar below each heatmap, comparisons in which the communities are more dissimilar are more red, whereas communities that are more similar are less red.

The observed richness (number of OTUs) across the individual samples ranged from 388 to 1833, with a mean of 1045. Rarefaction curves for individual tumor and mucin samples are depicted in Additional file 1: Figure S1, and indicate that further sampling would likely not significantly affect observed richness. While the number of OTUs varied between sampled body sites (tumor vs. mucin) in some patients, as a whole, the number of OTUs found in tumor tissue was not significantly different than that found in the free mucin (*P* = 0.24, Student’s *t*-test). As the microenvironment of tumor tissue may be quite different than free mucin, we tested for OTU enrichment based on tissue type (tumor vs. mucin) using the mothur implementation of Metastats [27]. Surprisingly, the number of sequences clustered into each OTU was not significantly different in either site, further highlighting the fact that the bacterial communities in the tumor and mucin tissue sites are highly similar. Furthermore, when we compared the differences between tumor and mucin community membership and relative abundance using the classic Jaccard and Yue & Clayton (thetaYC) indices, we found that the two communities were not significantly different (*P* = 0.947, AMOVA using Jaccard indices; *P* = 0.83, AMOVA using thetaYC indices). Given the apparent similarity between the tumor and mucin communities, we combined the sequence sets from the tumor and mucin for each patient (a combined total of 200,000 randomly sampled sequences from each patient). Using these combined datasets, we again compared the differences in community membership and relative abundance using the classic Jaccard and thetaYC indices, respectively. As shown in Figure 2, the membership of the communities in each patient displays a surprisingly high level of similarity. Between patients, the only differences seem to be in terms of relative abundance of community members (Figure 2B) rather than the types of taxa present (Figure 2A). For example, the thetaYC-based comparison between patients 141 and 144 (Figure 2B) indicates that the communities in these two patients differ in terms of the relative abundance of taxa present. This finding may be explained by community differences at both higher and lower taxonomic levels. As shown in Figure 1, at the phylum level there were more Proteobacteria present in the samples obtained from patient 141 than 144, whereas the number of Actinobacteria were greater in patient 144. At the genus level, patient 141 had approximately two times as many *Helicobacter* sp. compared to patient 144, and patient 144 had approximately 10 times as many *Propionibacterium* sp. compared to patient 141 (data not shown). Thus, differences in large taxonomic groups as well as individual taxons likely contribute to the community differences observed between PMP patients.

**Figure 3:**
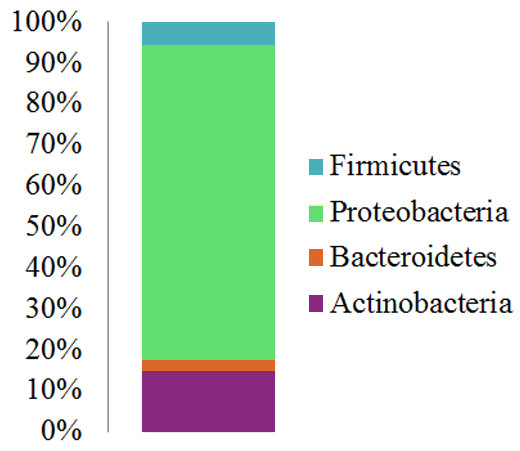
**Core set of sequences found in all PMP patients.** After combining the tumor and mucin sequence sets for each patient, sequences that were present in all patients were classified at a distance of 97%. The relative abundance of these sequences at the phylum level is shown.

Given the overall similarity between the tumor and mucin communities in a given patient we decided to use the combined tumor and mucin V6 sequence sets for each patient and asked whether there was a core set of sequences that were present in all patients. For a particular sequence to be considered as part of this core microbiome, we took a conservative approach and required that an exact match to a particular sequence be present in all patients. As shown in Figure 3, there were four phyla represented in the PMP core microbiome; the relative abundance of each phyla was similar to what was seen for the individual tumor and mucin samples, with the majority of sequences being classified as Proteobacteria (77%), followed by Actinobacteria (15%), Firmicutes (5.7%) and Bacteroidetes (2.3%). Within these phyla the core microbiome can be further broken down into 34 different groups that were classifiable to the genus level. A breakdown of this subset of sequences is shown in Table 2 and Additional file 1: Figures S2-S5, and a complete list of taxa identified in the PMP core microbiome is shown in Additional file 1: Table S1. While many of these taxa such as *Escherichia*, *Propionibacterium*, *Streptococcus* and *Helicobacter* are commonly found in humans, some of the taxa represented are more typically categorized as environmental organisms.

**Table 2:** PMP core microbiome sequences classifiable at the genus level

**Genus**	**Sequences**
Methylobacterium	106626
Variovorax	104621
Escherichia_Shigella	88823
Propionibacterium	81731
Pseudomonas	64037
Tessaracoccus	41711
Acinetobacter	35628
Helicobacter	33441
Streptococcus	17987
Acidovorax	15911
Moraxella	8777
Sphingomonas	6731
Methylotenera	6097
Haliscomenobacter	5883
Flavobacterium	5483
Polaromonas	5046
Brevibacillus	4674
Stenotrophomonas	4565
Methylophilus	4389
Diaphorobacter	4616
Limnohabitans	4453
Curvibacter	3149
Delftia	2904
Terrimonas	2587
Ralstonia	2372
Bdellovibrio	2288
Flectobacillus	1990
Arcicella	1904
Zoogloea	1663
Microbacterium	1306
Arcobacter	848
Pedobacter	834
Lactococcus	767
Roseomonas	633

### Direct detection of selected members of the PMP microbiome

**Figure 4:**
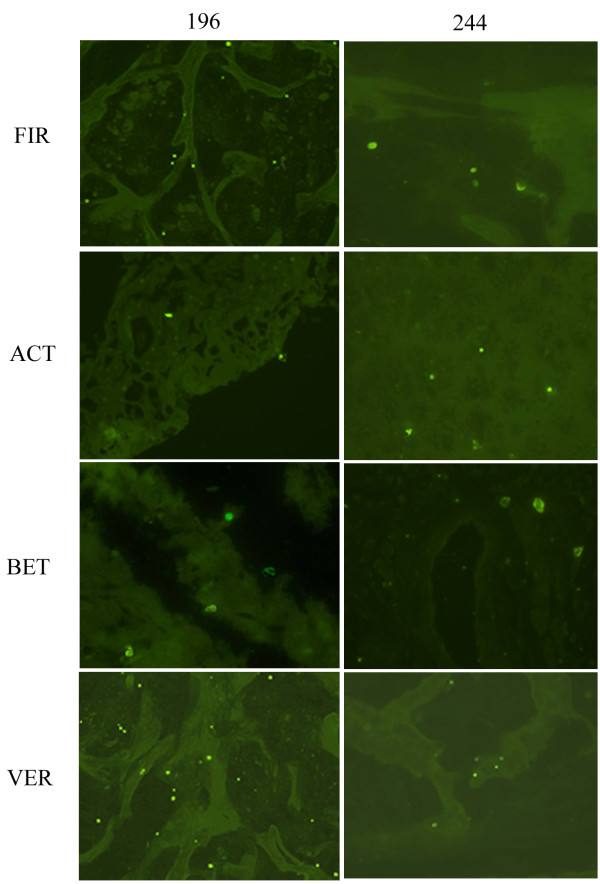
**Direct detection of bacterial taxa in PMP tumor tissue by ISH.** Hybridizations were carried out as described in the Materials and Methods. Using DNA probes complementary to taxon-specific rRNA sequences, we detected the presence of bacteria in additional patients not included in our sequencing study (patients 196 and 244 shown). Images were selected to show positive hybridization signal with a subset of probes used and are not intended to reflect relative abundance within tissue specimens. FIR, Firmicutes; ACT, Actinobacteria; BET, Betaproteobacteria; VER, Verrucomicrobiales.

As a complementary approach to our sequencing analysis, we used DNA probes complementary to taxon-specific regions of the 16S and 23S rRNAs to detect a subset of the taxa identified in the sequence-based analysis by *in situ* hybridization (ISH) (Table 1). For these experiments we utilized tissue sections from three patients that were included in the sequencing study (patients 135, 149 and 196) and two additional patients that were not included in the sequence-based analysis (patients 145 and 244). Figure 4 shows a representative subset of these results; hybridizations with the Firmicutes (FIR), Actinobacteria (ACT), Betaproteobacteria (BET), and Verrucomicrobiales (VER) probes in tissue sections from two PMP patients (196 and 244) are shown. At the phylum level we were able to detect Firmicutes, Bacteroidetes and Actinobacteria in tissue specimens from all five patients (Figure 4 and data not shown). Specifically within the Proteobacterial phylum, we detected the presence of Betaproteobacteria (Figure 4) and Gammaproteobacteria (data not shown); at the order level, we detected the presence of the commonly found human-associated Verrucomicrobiales (Figure 4), as well as the Rhizobiales (data not shown), which are commonly found within soil environments. Additionally, using genus-specific probes we verified the presence of *Pseudomonas*, *Propionibacterium* and *Streptococcus* sp., which are known human pathogens (data not shown). Combined, these data support our sequence-based analysis and confirm that members of these taxonomic groups are located within or directly associated with the PMP tissue.

### Isolation and identification of bacterial strains associated with PMP

**Figure 5:**
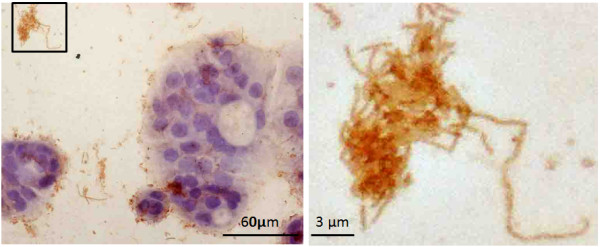
**Bacteria isolated from PMP tissue associates with MUC2.** Isolate PMP191F (an unclassified Chitinophagaceae) was cultured with MUC2 secreting HCT-29 cells. The rod shaped bacteria interact with cell-associated MUC2 (**left**) as well as secreted MUC2 (**right**). The positive reaction (seen as brown color) for MUC2 staining on the bacterial cells is consistent with interaction or adherence with MUC2. The image shown in the panel on the right is a magnification of the boxed region in the left panel.

To help us understand the impact that these bacteria have on disease, we isolated and characterized PMP-associated bacteria. Using microaerophilic conditions, we were able to successfully culture 11 isolates from eight patients. The identity of these bacterial isolates is shown in Table 3. *In vitro* assays using mucin secreting HT-29 cells indicated that at least some of these organisms are able to adhere to the mucin MUC2, which has been shown to be one of the mucins secreted by PMP cells. As an example, Figure 5 depicts the unclassified Chitinophagaceae strain interacting with secreted mucin. Importantly, one of the organisms we were able to culture from multiple patients, a *Propionibacterium* sp., was one of the more prevalent organisms identified in our core microbiome (Table 2) and directly detected by ISH (Figure 4). Taken together, these data further support our sequencing results and support a potential role for these bacteria in the maintenance or progression of PMP disease.

**Table 3:** Bacterial strains isolated from PMP tissue samples

**Isolate**	**Family and genus level classification**
PMP191F	unclassified Chitinophagaceae (100), Niastella (70)
PMP191M	Bradyrhizobiaceae (100), Bosea (100)
PMP191C	Dermacoccaceae (100), Dermacoccus (100)
PMP196	Propionibacteriaceae (100), Propionibacterium (100)
PMP213	Propionibacteriaceae (100), Propionibacterium (100)
PMP215	Pseudonocardiaceae (100), Amycolatopsis (100)
PMP219	Propionibacteriaceae (100), Propionibacterium (100)
PMP229	Propionibacteriaceae (100), Propionibacterium (100)
PMP238	Corynebacteriaceae (100), Corynebacterium (100)
PMP267-3	Propionibacteriaceae (100), Propionibacterium (100)
PMP267B	Corynebacteriaceae (100), Corynebacterium (100)

Sequences were amplified using the 8F and 1492R primers, sequenced using the 907R and/or 1492R primers and classified using the RDP classifier; % bootstrap support for each classification is shown in parentheses.

### Antibiotic treatment efficacy

**Figure 6:**
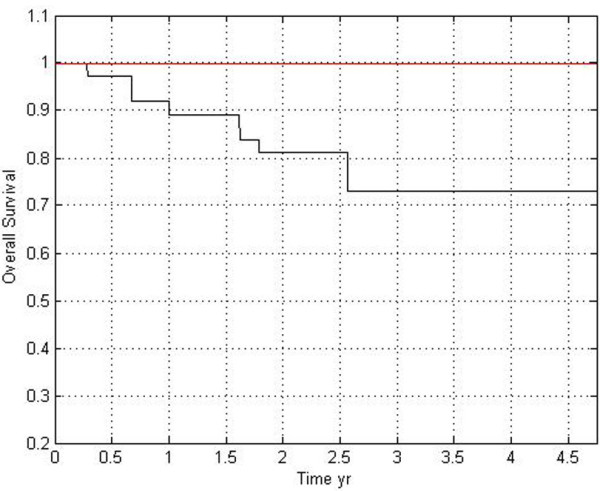
**Patient survival after antibiotic treatment.** Survival of PMP LN negative PMCA patients with or without antibiotic treatment; the red line indicates survival of patients that were treated with antibiotics (n = 6), whereas the black line indicates patients that were not given this treatment (n = 37). Differences in percent survival were compared using a Log-rank statistical test, *P* = 0.078.

Having identified bacteria associated with PMP, we next tested whether antibiotic treatment could serve as an additional treatment option for patients. To this end, we designed a pilot clinical study to evaluate the efficacy of antibiotic treatment in PMP patients. Since *H*. *pylori* was previously identified in PMP tissue [10], the initial treatment regimen was designed to treat this organism. Starting in September 2007, a test group of 21 PMP patients was treated with antibiotics in addition to cytoreductive surgery and hyperthermic intraperitoneal chemotherapy using mitomycin. The antibiotic regimen used was the Prevpac®, which includes lansoprazole, amoxicillin, and clarithromycin, taken twice daily for 10–14 days. Seventeen of the patients received two courses of antibiotics, one course three weeks prior to surgery and a second course six to eight weeks following surgery. The four additional patients only received one course prior to surgery, since they had difficulty in tolerating the antibiotics used. The antibiotic treatment group consisted of eight DPAM patients, 11 PMCA patients, one patient with both diagnoses (from separate biopsies), and one patient where the diagnosis was not recorded. Based on a recent study, which indicated that lymph node (LN) status is a good indicator of survival in PMP, we divided the test patients into two major groups based on whether they were LN positive or negative. Thirteen of the patients in the antibiotic treatment group were LN negative and seven patients were LN positive. One patient did not receive a LN assessment. Initially we compared the survival of the LN negative PMP patients that received the antibiotic treatment to a group of LN negative patients that had not received the PrevPac® treatment regimen. This non-antibiotic control group was part of a larger cohort of PMP patients that are currently being followed. Overall, the LN negative patients who received antibiotics showed a higher percent survival than has been previously reported (9); when compared to the group of LN negative non-antibiotic treated patients, the difference in survival was statistically significant (*P* < 0.05, Log-rank test). However, we noted that this comparison included antibiotic treated patients with both DPAM and PMCA diagnosis (six PMCA and seven DPAM), whereas patients in the control group only had PMCA. In order to make an assessment of the antibiotic treatment efficacy that took diagnosis into account, we next compared survival rates of only LN negative patients with a PMCA diagnosis; we compared the survival of the six LN negative PMCA patients in the antibiotic treatment group to a cohort of 37 LN negative PMCA patients that had not received therapy (Figure 6). As described above, patients in this control group are part of a larger group of PMP patients currently being followed (described in (9)). The difference in survival between these two LN negative PMCA patient groups did not reach statistical significance (*P* = 0.078, Log-rank test); all of the patients that received antibiotics survived, whereas only ~70% of those that did not receive antibiotics survived. Even though this value is not statistically significant, it does indicate a trend that suggests that antibiotics could be beneficial. Though not included in that comparison, it is important to note that all seven DPAM patients who received antibiotics are also surviving as of February, 2013. Specifically, our results suggest that antibiotics may be beneficial in treating LN negative PMP patients, and that elimination of bacteria before PMP has had a chance to metastasize can extend overall survival time of LN negative PMP patients. Importantly, the survival rate of LN positive patients within the pilot group that received antibiotic therapy was effectively the same as those that did not receive antibiotics (data not shown). Importantly, an additional recent study from our group showed that antibiotic treatment significantly reduced the number of bacteria found in PMP tissue [28]. Thus, *en masse* our results support our hypothesis that eliminating bacteria from the affected peritoneal tissue prior to metastasis may improve survival of patients with PMP disease.

## Discussion

PMP is a rare but serious disease that results in significant morbidity and mortality. As patient outcome is frequently more negative once the disease progresses to the more severe form (PMCA), PMP is most effectively treated prior to this transition. However, despite early intervention, PMP patients experience frequent relapses and the long-term prognosis for PMP patients is dismal, with only ~30% surviving past 10 years. As such there is still a great need for novel or supplementary treatment options that will increase patient survival.

A previous study highlighted the fact that bacteria are present within the peritoneum of PMP patients, but not in the patients that did not have PMP [10]. Because the peritoneum is typically considered to be sterile, this finding was pursued in greater detail. In the current study, we identified a core set of bacteria that are present in PMP tumor tissue and free mucin. The majority of the taxa present in this group were classified as Proteobacteria, and the overall profile of the core microbiome (Figure 3) reflects that of the individual samples (Figure 1). Across the 11 patients in our study, the major difference in the community structures was related to the relative abundance of bacterial taxa. These differences can be seen at both the phylum and lower taxonomic levels. Interestingly, the dominant phylum present was the Proteobacteria; while this group is commonly found in and on the human body [11,13,18,19], virtually none of the healthy subject microbiomes described to date are dominated by this phylum. On the other hand, Proteobacteria are a dominant member of the respiratory tract microbiome associated with cystic fibrosis (CF) patients [20,29,30]. The abundance of Proteobacteria in both of these disease states is perhaps the result of similarities in the CF lung environment and PMP tissue in the peritoneum. In both CF and PMP, there is an abundance of mucin/mucus secretion, which may enrich for the Proteobacteria as well as some of the typically “environmental” bacteria found in our sequence-based study (Table 2 and Additional file 1: Table S1). While the possibility exists that these bacteria may directly utilize mucin or affect mucin secretion, currently the exact role of these bacteria in stimulating mucin production in this disease is unknown.

In addition to the dominance of the Proteobacterial phylum, the PMP core microbiome also shares some additional characteristics with that of CF airways. Specifically, there are several non-Proteobacterial genera that are present in both microbiomes. As shown in Table 2 and Additional file 1: Table S1, of those classifiable at the genus level, one of the more prevalent groups detected in our study was the *Streptococcus* sp. Members of this genus, which includes the *S*. *milleri* group (SMG), have been recently shown to be a prominent part of CF disease [20] and are known to be associated with several types of human disease [31-34]. Furthermore, other *Streptococcus* sp. have been shown to increase virulence of other bacterial pathogens such as *P*. *aeruginosa*[35], which could be found in PMP tissue given that members of the *Pseudomonas* genus were found in the PMP core microbiome (Table 2 and Additional file 1: Table S1). A previous study identified the carcinogenic bacterium *H*. *pylori* in PMP tissue samples and determined that the density of *H*. *pylori* correlated to PMP disease severity [10]. Consistent with this study, we also detected the presence of *Helicobacter* sp. in the PMP core microbiome (Table 2 and Additional file 1: Table S1). While the precise role of these bacteria in PMP disease is unclear, *H*. *pylori* is known to induce a pro-inflammatory response [36] and secrete an oncogenic virulence factor [37] that may contribute to carcinogenesis and/or the maintenance of PMP disease. While our study design did not allow us to confidently make species level taxonomic classification, the presence of *Streptococcus* sp., *Pseudomonas* sp., *Helicobacter* sp. and other taxonomic groups that contain known human pathogens in the PMP core microbiome highlights the possibility that these bacteria play a role in PMP disease and certainly merits more detailed study.

One attractive feature of performing sequence-based bacterial community analyses is the ability to identify possible keystone taxonomic groups or species that are enriched in a particular body site or disease state. Recently, this feature was exploited to identify *Fusobacterium* sp. as being enriched in patients with colorectal cancer [12,17]. However, unlike those studies, for our work, there did not appear to be any one bacterial type that was enriched in either of the two sampling sites (tumor and mucin), or in one disease state versus the other (DPAM vs PCMA). This finding perhaps suggests that it is the combination of many bacterial types present (*i*.*e*. the community structure) that is important in PMP rather than a single keystone species and highlights the importance of studying these organisms as a community in addition to studies focused on single bacterial types. However, we do note that as shown in Figure 1, there are particular taxa that are more abundant in some patients than others; as a result, we cannot completely rule out the possibility that the increased abundance of one particular taxon/species could have an effect on disease progression. As highlighted in recent study of colorectal cancer [38], it is possible that as our understanding of PMP-associated bacteria evolves, so will our understanding of the role of particular taxa in the disease.

In an effort to characterize PMP-associated organisms, we have begun to culture bacteria from PMP samples. To date, we have successfully cultured several organisms from multiple patients. Among those bacteria cultured (Table 3), the *Propionibacterium* sp. was isolated most frequently. Comparison of these 16S sequences to completed genomes in GenBank indicates that these *Propionibacterium* sp. are likely to be *P*. *acnes*. *P*. *acnes* is commonly found on human skin and has been implicated in several types of human diseases including endopthalmitis and bone infections. Interestingly, a recent ISH-based study found *P*. *acnes* in ~80% of cancerous prostates [39]. In that study, the authors were also able to culture *P*. *acnes* from cancerous prostates and showed that incubation with this bacterium elicited a strong pro-inflammatory response in host cells [39], which may be a contributing factor to disease progression. The potential role for the other cultured isolates is less clear. However, *in vitro* experiments with the unclassified Chitinophagaceae isolate PMP191F indicate that this organism does interact with the PMP-associated mucin, MUC2 (Figure 5).

The presence of a consistent group of bacteria in all PMP patients brings to light one distinct possibility: if these bacteria do play a role in the maintenance of PMP disease or PMP progression, then treatments aimed at clearing this polymicrobial infection should improve patient outcome. We tested this hypothesis in a pilot clinical study using new patients as well as existing patient data. We found that treating PMP patients with a combination of antibiotics (Prevpac®) improved overall survival. While these pilot study findings are important in that they strongly suggest that antibiotics may be a viable supplementary treatment for PMP, additional patients need to be enrolled in a larger clinical study to confirm the positive effect of antibiotic treatment on PMP disease. Future work will focus on optimizing specimen transport and culture methods to increase the recovery of bacterial isolates from PMP tissue, and on developing an animal model of PMP, which will allow us to better understand the role of individual bacterial species in PMP disease. Once we have gained a more complete understanding of the role each bacterial taxon plays in PMP, antibiotic therapy could be tailored to target specific taxa in a particular patient.

The study presented herein characterized a previously unknown aspect of PMP: the PMP-associated microbiome. As a result of our sequence-based analysis, direct detection of bacterial taxa by ISH and culturing efforts, we have identified a possible novel avenue for treating this deadly disease. While these findings identify a conserved microbiome and show that antibiotic treatment extends patient survival, more work is needed to better understand the role of bacterial communities in PMP disease and the benefits gained from eliminating or reducing the load of these bacteria. Finally, this study also highlights the possibility that other diseases not currently known to be linked to the presence of bacteria may in fact be associated with bacterial infection.

## Competing interests

The authors declare that they have no competing interests.

## Authors’ contributions

JG contributed to study design, performed the sequence-based bacterial community analysis, analyzed data, and wrote the manuscript; CSM performed the ISH experiments; CF contributed to the sequence-based bacterial community analysis and wrote custom JAVA programs; HL contributed to sample processing; KB contributed to sequence-based study design; TM contributed to study design and data analysis; JF contributed to sample processing and study design; CN contributed to study design; AS contributed to study design, performed surgical procedures on PMP patients and analyzed data; AD contributed to study design and analyzed data; DL and AC contributed to study design; TT contributed to study design, performed bacterial culturing from PMP tissue, and analyzed data; DM contributed to study design, analyzed data, and wrote the manuscript. All authors read and approved the final manuscript.

## Supplementary Material

Additional file 1: Figure S1 Rarefaction analysis of individual Tumor and Mucin samples, **Figures S2-S5**-**Figure S2**: Breakdown of PMP core microbiome that were classifiable at the genus level, and **Table S1**: Complete breakdown of sequences in the PMP core microbiome.” Click here for file
