# Supplementary material for: A core microbiome associated with the peritoneal tumors of pseudomyxoma peritonei
Source: Orphanet J Rare Dis. 2013 Jul 12;8:105. doi: 10.1186/1750-1172-8-105 (PMC3729368; doi:10.1186/1750-1172-8-105)
Supplement: Additional file 1: Figure S1 — Rarefaction analysis of individual Tumor and Mucin samples, Figures S2-S5-Figure S2: Breakdown of PMP core microbiome that were classifiable at the genus level, and Table S1: Complete breakdown of sequences in the PMP core microbiome.” [file 1750-1172-8-105-S1.pdf]

Supplemental information

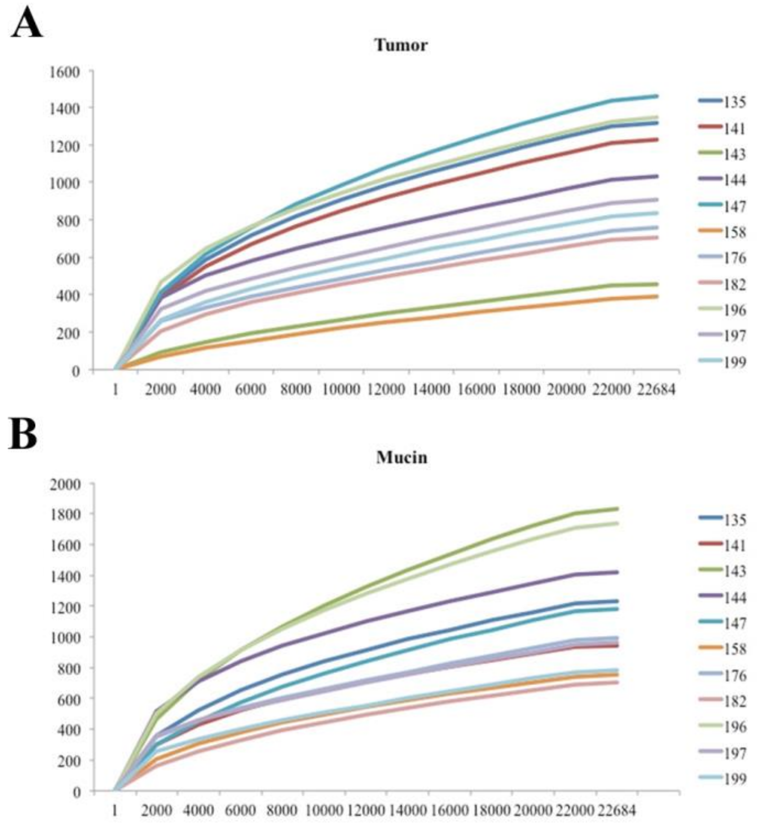

Figure S1. Rarefaction analysis of individual Tumor (A) and Mucin (B) samples. Normalized observed number of OTUs are plotted against sampling depth. Sample numbers indicate patient designations.

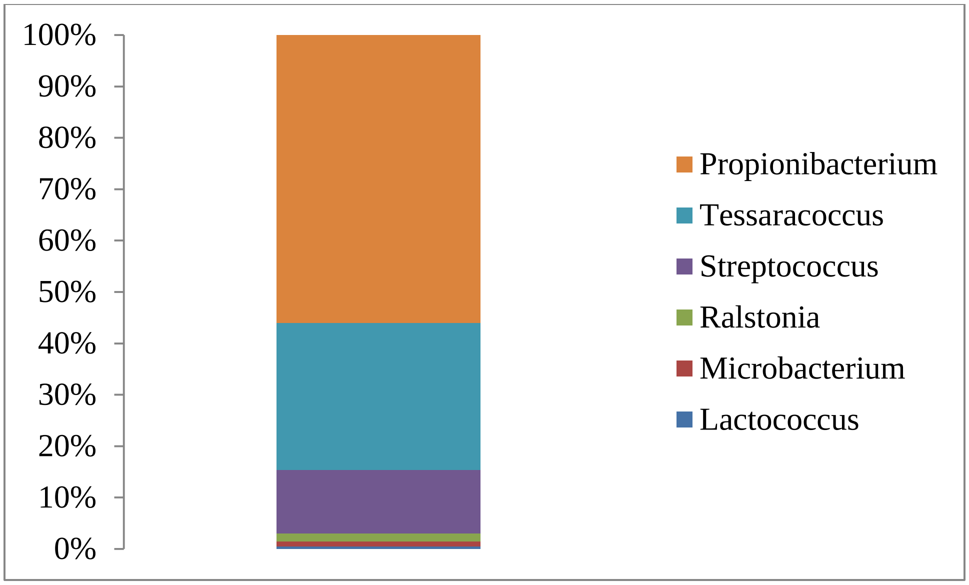

Figure S2. Breakdown of PMP core microbiome Actinobacteria that were classifiable at the genus level (Table 1). The percent of total Actinobacteria is shown for each taxon.

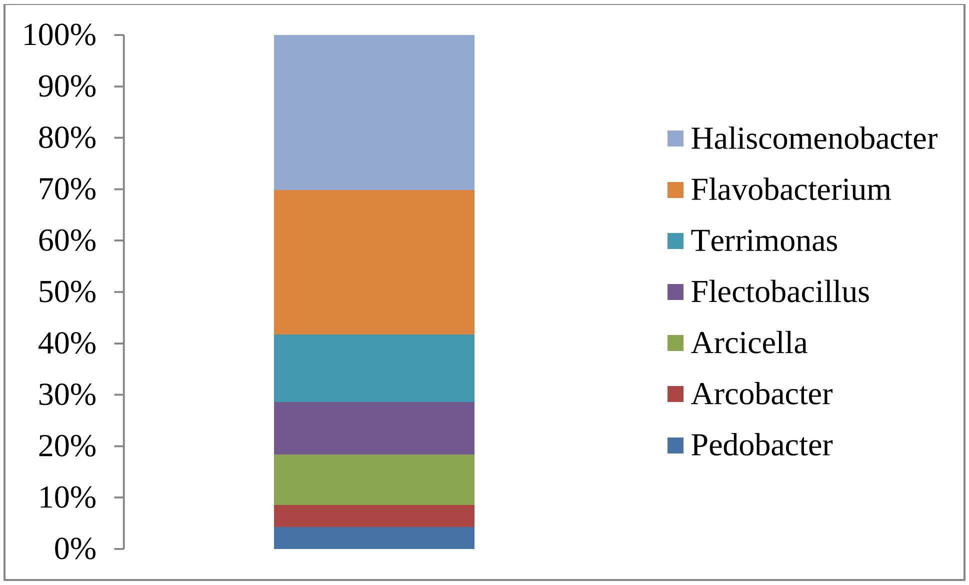

Figure S3. Breakdown of PMP core microbiome Bacteroidetes that were classifiable at the genus level (Table 1). The percent of total Bacteroidetes is shown for each taxon.

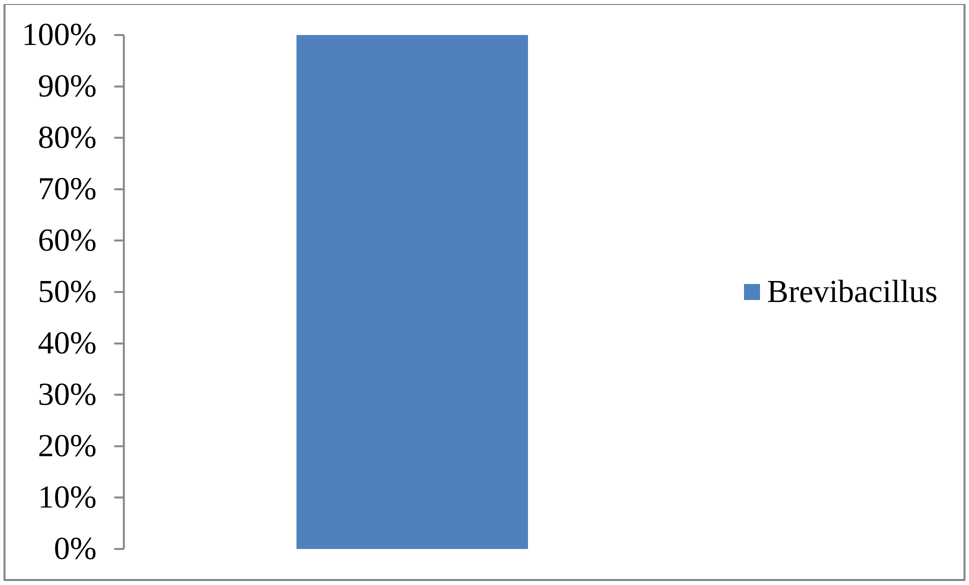

Figure S4. Breakdown of PMP core microbiome Firmicutes that were classifiable at the genus level (Table 1). *Brevibacillus* was the only Firmicute taxon identified at this level.

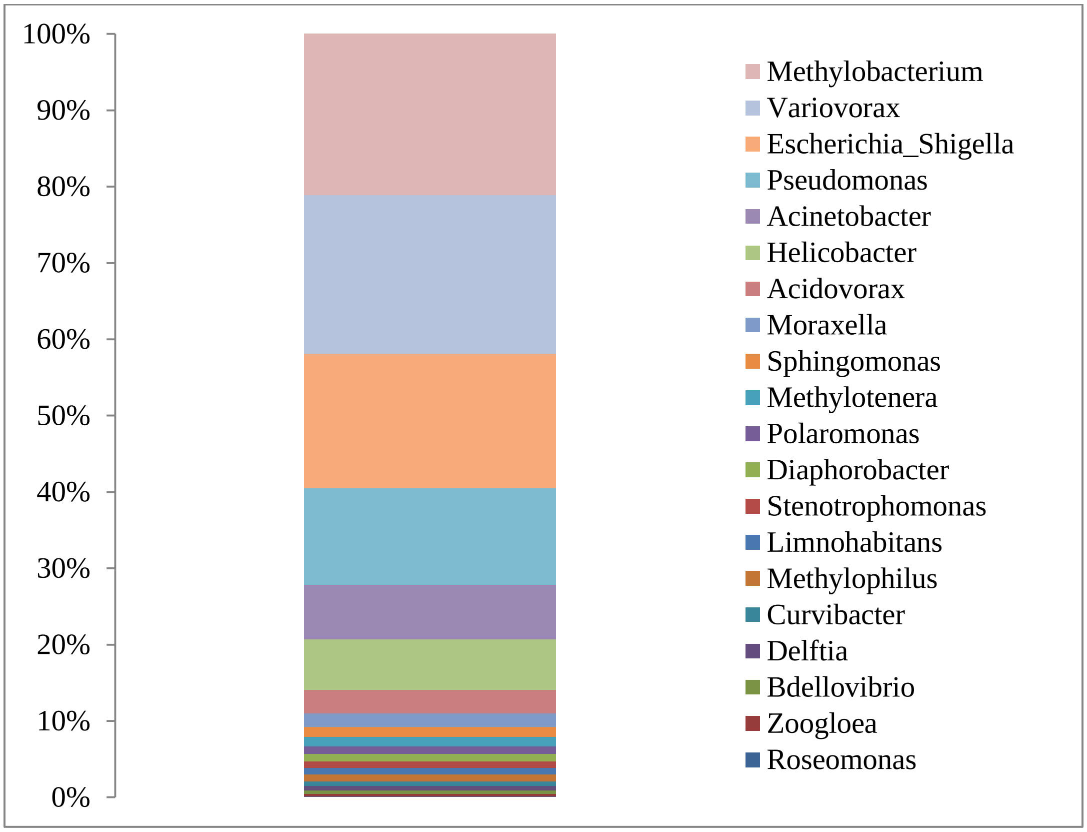

Figure S5. Breakdown of PMP core microbiome Proteobacteria that were classifiable at the genus level (Table 1). The percent of total Proteobacteria is shown for each taxon.

Table S1. Complete breakdown of sequences in the PMP core microbiome

			PMP Patient										
taxlevel	taxon	total seqs	135	141	143	144	147	158	176	182	196	197	199
1	Bacteria	941151	44786	101360	95784	71326	92474	141553	70443	109509	68398	63308	82210
2	Actinobacteria	142834	3519	2439	16176	18766	4368	4596	19882	11693	20828	19774	20793
3	Actinobacteria	142834	3519	2439	16176	18766	4368	4596	19882	11693	20828	19774	20793
4	Actinomycetales	142834	3519	2439	16176	18766	4368	4596	19882	11693	20828	19774	20793
5	Microbacteriaceae	19392	624	403	14687	552	951	127	584	74	655	439	296
6	Microbacterium	1306	51	184	140	132	235	3	179	1	144	175	62
6	unclassified	18086	573	219	14547	420	716	124	405	73	511	264	234
5	Propionibacteriaceae	123442	2895	2036	1489	18214	3417	4469	19298	11619	20173	19335	20497
6	Propionibacterium	81731	893	1373	440	14357	1027	1912	14289	5331	15151	15135	11823
6	Tessaracoccus	41711	2002	663	1049	3857	2390	2557	5009	6288	5022	4200	8674
2	Bacteroidetes	21145	982	3299	1416	2730	5472	198	1875	300	2164	1720	989
3	Sphingobacteria	15662	655	3029	878	1479	5145	159	1263	246	1361	846	601
4	Sphingobacteriales	15662	655	3029	878	1479	5145	159	1263	246	1361	846	601
5	Saprospiraceae	5883	116	1998	94	159	2979	11	147	75	152	1	151
6	Haliscomenobacter	5883	116	1998	94	159	2979	11	147	75	152	1	151
5	Chitinophagaceae	2587	66	564	76	160	1341	33	57	17	111	37	125
6	Terrimonas	2587	66	564	76	160	1341	33	57	17	111	37	125
5	Cytophagaceae	3894	147	46	307	660	204	83	664	55	840	697	191
6	Arcicella	1904	45	26	148	315	181	46	229	22	437	350	105
6	Flectobacillus	1990	102	20	159	345	23	37	435	33	403	347	86
5	Sphingobacteriaceae	834	2	9	41	141	3	14	305	44	211	49	15
6	Pedobacter	834	2	9	41	141	3	14	305	44	211	49	15
5	unclassified	2464	324	412	360	359	618	18	90	55	47	62	119
6	unclassified	2464	324	412	360	359	618	18	90	55	47	62	119
3	Flavobacteria	5483	327	270	538	1251	327	39	612	54	803	874	388
4	Flavobacteriales	5483	327	270	538	1251	327	39	612	54	803	874	388
5	Flavobacteriaceae	5483	327	270	538	1251	327	39	612	54	803	874	388
6	Flavobacterium	5483	327	270	538	1251	327	39	612	54	803	874	388
2	Proteobacteria	721710	29328	91740	74200	45311	63133	135968	45524	96214	42619	39604	58069
3	Alphaproteobacteria	115437	761	27438	9287	9690	18950	13725	3346	23313	3467	2802	2658
4	Rhizobiales	106626	590	27232	6369	7740	18704	13384	2604	23141	2437	2350	2075
5	Methylobacteriaceae	106626	590	27232	6369	7740	18704	13384	2604	23141	2437	2350	2075
6	Methylobacterium	106626	590	27232	6369	7740	18704	13384	2604	23141	2437	2350	2075
4	Rhodospirillales	633	4	29	7	48	4	33	144	10	185	83	86
5	Acetobacteraceae	633	4	29	7	48	4	33	144	10	185	83	86
6	Roseomonas	633	4	29	7	48	4	33	144	10	185	83	86
4	Sphingomonadales	8178	167	177	2911	1902	242	308	598	162	845	369	497
5	Sphingomonadaceae	8178	167	177	2911	1902	242	308	598	162	845	369	497
6	Sphingomonas	6731	146	113	2910	1685	216	182	497	120	550	176	136
6	unclassified	1447	21	64	1	217	26	126	101	42	295	193	361
3	Betaproteobacteria	275417	4035	51676	8729	17192	34982	31766	12777	63928	13780	12375	24177
4	Burkholderiales	255953	3237	47179	8563	15417	30563	31588	11977	63198	12289	9603	22339
5	Burkholderiaceae	72253	355	39980	763	3400	21647	540	1124	980	1198	756	1510
6	Ralstonia	2372	58	306	153	725	137	180	127	174	181	121	210
6	unclassified	69881	297	39674	610	2675	21510	360	997	806	1017	635	1300
5	Comamonadaceae	145981	2241	3307	6624	9161	4929	30567	6872	61332	9697	7173	4078
6	Acidovorax	15911	660	1752	3650	1800	2866	344	619	681	1483	1069	987
6	Curvibacter	3149	267	186	451	665	326	225	218	16	300	303	192
6	Delftia	2904	32	14	21	623	17	89	1186	9	565	186	162
6	Diaphorobacter	4616	41	1	22	193	7	153	618	478	2296	44	763
6	Limnohabitans	4453	314	378	679	1238	435	174	228	98	399	325	185
6	Polaromonas	5046	144	109	193	752	136	417	285	419	1219	1076	296
6	Variovorax	104621	575	662	1361	3236	865	28999	2689	59409	2787	2947	1091
6	unclassified	5281	208	205	247	654	277	166	1029	222	648	1223	402
5	unclassified	37719	641	3892	1176	2856	3987	481	3981	886	1394	1674	16751
6	unclassified	37719	641	3892	1176	2856	3987	481	3981	886	1394	1674	16751
4	Hydrogenophilales	2865	17	74	2	445	12	6	170	223	276	816	824
5	Hydrogenophilaceae	2865	17	74	2	445	12	6	170	223	276	816	824
6	Hydrogenophilus	2865	17	74	2	445	12	6	170	223	276	816	824
4	Methylophilales	10486	587	2941	101	1043	2883	7	312	197	793	1404	218
5	Methylophilaceae	10486	587	2941	101	1043	2883	7	312	197	793	1404	218
6	Methylophilus	4389	495	499	40	628	301	1	279	145	628	1224	149
6	Methylotenera	6097	92	2442	61	415	2582	6	33	52	165	180	69
4	Rhodocyclales	1663	34	49	10	28	101	103	206	195	141	159	637
5	Rhodocyclaceae	1663	34	49	10	28	101	103	206	195	141	159	637
6	Zoogloea	1663	34	49	10	28	101	103	206	195	141	159	637
4	unclassified	4450	160	1433	53	259	1423	62	112	115	281	393	159
5	unclassified	4450	160	1433	53	259	1423	62	112	115	281	393	159
6	unclassified	4450	160	1433	53	259	1423	62	112	115	281	393	159

Table S1 (cont.)

	taxon	total seqs	PMP Patient 135	141	143	144	147	158	176	182	196	197	199
3	Deltaproteobacteria	2288	12	148	15	283	59	93	354	106	569	448	201
4	Bdellovibrionales	2288	12	148	15	283	59	93	354	106	569	448	201
5	Bdellovibrionaceae	2288	12	148	15	283	59	93	354	106	569	448	201
6	Bdellovibrio	2288	12	148	15	283	59	93	354	106	569	448	201
3	Epsilonproteobacteria	34289	27	6990	975	3467	653	434	4945	521	4355	6873	5049
4	Campylobacterales	34289	27	6990	975	3467	653	434	4945	521	4355	6873	5049
5	Campylobacteraceae	848	11	5	69	142	55	47	138	33	163	57	128
6	Arcobacter	848	11	5	69	142	55	47	138	33	163	57	128
5	Helicobacteraceae	33441	16	6985	906	3325	598	387	4807	488	4192	6816	4921
6	Helicobacter	33441	16	6985	906	3325	598	387	4807	488	4192	6816	4921
3	Gammaproteobacteria	285025	24403	2251	55089	14108	5078	89765	23757	8058	19987	16786	25743
4	Enterobacteriales	107398	693	465	656	1829	1093	88561	3155	1904	1886	2270	4886
5	Enterobacteriaceae	107398	693	465	656	1829	1093	88561	3155	1904	1886	2270	4886
6	Escherichia_Shigella	88823	302	343	360	1660	417	73796	2583	1528	1576	2012	4246
6	unclassified	18575	391	122	296	169	676	14765	572	376	310	258	640
4	Alteromonadales	42103	17755	216	142	2208	282	208	3965	1447	4887	3009	7984
5	Alteromonadaceae	42103	17755	216	142	2208	282	208	3965	1447	4887	3009	7984
6	unclassified	42103	17755	216	142	2208	282	208	3965	1447	4887	3009	7984
4	Chromatiales	424	79	17	17	2208	6	60	1	31	15	107	37
5	unclassified	424	79	17	17	2208	6	60	1	31	15	107	37
6	unclassified	424	79	17	17	2208	6	60	1	31	15	107	37
4	Pseudomonadales	108442	4697	795	53329	2208	2892	559	13472	3489	8495	5474	9205
5	Moraxellaceae	44405	1338	538	839	2208	399	211	12912	2420	7711	4917	8382
6	Acinetobacter	35628	1334	359	832	2208	392	190	11835	2365	6521	3361	4867
6	Moraxella	8777	4	179	7	2208	7	21	1077	55	1190	1556	3515
5	Pseudomonadaceae	64037	3359	257	52490	2208	2493	348	560	1069	784	557	823
6	Pseudomonas	64037	3359	257	52490	2208	2493	348	560	1069	784	557	823
4	Xanthomonadales	4565	67	145	11	2208	88	103	1259	240	951	345	475
5	Xanthomonadaceae	4565	67	145	11	2208	88	103	1259	240	951	345	475
6	Stenotrophomonas	4565	67	145	11	2208	88	103	1259	240	951	345	475
4	unclassified	22093	1112	613	934	2208	717	274	1905	947	3753	5581	3156
5	unclassified	22093	1112	613	934	2208	717	274	1905	947	3753	5581	3156
6	unclassified	22093	1112	613	934	2208	717	274	1905	947	3753	5581	3156
3	unclassified	9254	90	3237	105	2208	3411	185	345	288	461	320	241
4	unclassified	9254	90	3237	105	2208	3411	185	345	288	461	320	241
5	unclassified	9254	90	3237	105	2208	3411	185	345	288	461	320	241
6	unclassified	9254	90	3237	105	2208	3411	185	345	288	461	320	241
2	Firmicutes	53508	10849	3640	3887	4112	19375	730	2815	1229	2513	2147	2211
3	Bacilli	39109	5488	2116	1704	2812	17558	699	1531	1067	2123	2053	1958
4	Bacillales	13232	4716	1855	1344	1609	1706	265	213	283	256	307	678
5	Paenibacillaceae_1	4674	880	726	13	1344	21	204	181	275	205	216	609
6	Brevibacillus	4674	880	726	13	1344	21	204	181	275	205	216	609
5	unclassified	8558	3836	1129	1331	265	1685	61	32	8	51	91	69
6	unclassified	8558	3836	1129	1331	265	1685	61	32	8	51	91	69
4	Lactobacillales	20614	622	142	307	662	14443	320	880	586	992	770	890
5	Lactobacillaceae	777	140	5	35	47	119	31	73	59	116	80	72
6	Lactobacillus	777	140	5	35	47	119	31	73	59	116	80	72
5	Streptococcaceae	18754	434	112	217	583	14191	262	582	438	744	506	685
6	Lactococcus	767	38	29	14	98	58	18	122	29	251	109	1
6	Streptococcus	17987	396	83	203	485	14133	244	460	409	493	397	684
5	unclassified	1083	48	25	55	32	133	27	225	89	132	184	133
6	unclassified	1083	48	25	55	32	133	27	225	89	132	184	133
4	unclassified	5263	150	119	53	541	1409	114	438	198	875	976	390
5	unclassified	5263	150	119	53	541	1409	114	438	198	875	976	390
6	unclassified	5263	150	119	53	541	1409	114	438	198	875	976	390
3	unclassified	14399	5361	1524	2183	1300	1817	31	1284	162	390	94	253
4	unclassified	14399	5361	1524	2183	1300	1817	31	1284	162	390	94	253
5	unclassified	14399	5361	1524	2183	1300	1817	31	1284	162	390	94	253
6	unclassified	14399	5361	1524	2183	1300	1817	31	1284	162	390	94	253
